# Unrestricted kinematic alignment for total knee arthroplasty: a mid- to long-term follow-up non-designer single surgeon study with survival analysis

**DOI:** 10.1302/2633-1462.75.BJO-2025-0399.R1

**Published:** 2026-05-12

**Authors:** Francesco Mancuso, Khaled Al-Mohamadi, Christian Kleinert, Stijn Cornelissen, Hemant Pandit, Dragan Jeremic

**Affiliations:** 1 Orthopaedics Clinic, Department of Medicine, University of Udine, ASUFC, Piazzale Santa Maria della Misericordia, Udine, Italy; 2 Arthroplasty Center, St. Vicenz Hospital, Brakel, Germany; 3 Leeds Institute of Rheumatic and Musculoskeletal Medicine, University of Leeds, Chapel Allerton Hospital, Leeds, UK

**Keywords:** Total knee arthroplasty, Kinematic alignment, Unrestricted, Survival, Functional outcome, total knee arthroplasties (TKAs), Knees, Forgotten Joint Score (FJS), valgus, Knee Injury and Osteoarthritis Outcome Score (KOOS), reoperations, patient-reported outcome measures (PROMs), hip, NRS, aseptic loosening

## Abstract

**Aims:**

Kinematic alignment (KA) offers a personalized approach by restoring each patient’s pre-arthritic joint lines. Unrestricted KA (urKA) removes alignment boundaries defined by restricted KA concept, but concerns regarding reproducibility and clinical success persist. This study evaluates mid- to long-term survivorship and functional outcomes of urKA total knee arthroplasty (urKA-TKA) performed using a manual surgical technique by a single surgeon, with a minimum follow-up of three years.

**Methods:**

We prospectively reviewed 229 consecutive urKA-TKAs performed between March 2014 and October 2019 using a medial pivot design TKA and calipered measured resection (mean follow-up 87 months (36 to 136)). Patella was not routinely resurfaced. No restrictions were applied regarding preoperative limb alignment. Outcomes included survivorship (revision and reoperation), radiological alignment, and patient-reported outcomes: Knee Injury and Osteoarthritis Outcome Score (KOOS), Forgotten Joint Score (FJS), and Numerical Rating Scale (NRS) for pain. Survivorship was calculated using Kaplan-Meier analysis.

**Results:**

Preoperative and postoperative hip-knee-ankle (HKA) axis ranged respectively from -20.4° to 25.2° and -9.4° to 10.6°. The mean KOOS improved from 28.8 (SD 10.3) preoperatively to 75.8 (SD 16.6) postoperatively (p < 0.001). FJS was 87.6 (SD 17.7) at final follow-up, while NRS pain scores averaged 1.6 (SD 2.4), with 60% reporting no pain. At nine years (n = 32 at risk), implant survivorship was 97% free from revision for any reason and 96% free from reoperation, with no cases of aseptic loosening. Patients with preoperative alignment outliers (>± 5° HKA) demonstrated greater improvements in KOOS and FJS than neutral knees. Outcomes and implant survival were similar between knees within or outside restricted alignment boundaries. A trend towards higher rates of secondary patellar resurfacing was observed in valgus phenotypes, although this finding was not statistically significant.

**Conclusion:**

UrKA TKA provides excellent mid- to long-term survivorship and significant functional improvement without compromising safety in all knee phenotypes including patients with extreme preoperative alignment.

Cite this article: *Bone Jt Open* 2026;7(5):627–635.

## Introduction

Personalized alignment strategies have been developed to address the limitations associated with traditional mechanical alignment (MA).^1^ Among these, kinematic alignment (KA) aims to restore the patient’s native knee anatomy by re-establishing the three principal kinematic axes, usually avoiding ligament releases and potentially improving the perception of a more natural joint of the knee.^2^ Howell et al^2^ first introduced unrestricted KA (urKA), which reproduces native alignment without predefined boundaries. However, native lower-limb and knee anatomies vary widely, with approximately 40% of patients demonstrating alignment patterns that deviate from mechanical neutrality.^3^ Such deviations have raised concerns regarding abnormal wear patterns and implant longevity.

To mitigate these issues, restricted KA (rKA) was proposed as a hybrid approach between MA and urKA. This strategy introduces alignment boundaries to avoid extreme outliers, commonly targeting an arithmetic hip-knee angle within 3° of neutral and limiting joint line obliquity (JLO) to 5°.^4^ JLO reflects the orientation of the knee joint line and is derived from standard radiological alignment parameters. Additional personalized concepts have since emerged, including functional alignment, which optimizes component positioning based on intraoperative ligament balance balance,^5^ and inverse KA,^6^ which prioritizes restoration of native tibial anatomy followed by femoral adjustments to balance flexion and extension gaps.

Despite these innovations, current evidence, including a recent meta-analysis of over 3,000 total knee arthroplasties (TKAs), demonstrates no significant differences in revision or complication rates between alignment strategies. While KA-TKA may yield improved patient-reported outcome measures (PROMs) and range of motion (ROM), these benefits appear marginal, and concerns remain regarding reproducibility, patellofemoral kinematics, and long-term implant survival.

Several meta-analyses comparing KA and MA in total knee arthroplasty have reported modest improvements in PROMs and postoperative ROM with KA, with limited clinical relevance^7^ and without significant differences in complication or revision rates.^8^ However, the clinical relevance of these findings appears limited, and the overall quality of evidence remains moderate, with substantial heterogeneity in surgical techniques, implants, and outcome reporting. Importantly, most available data are restricted to short- to mid-term follow-up and frequently derive from designer series, particularly those reported by Howell et al.^9,10^ Although long-term studies on urKA suggest acceptable implant survivorship, non-designer long-term evidence remains scarce.^11^ The lack of boundaries for urKA raises concerns about reproducibility, impact on patella-femoral joint kinematics, and long-term implant survival.^12^

KA aims to restore each patient’s pre-arthritic joint lines and has shown promising clinical results; however, the urKA approach, which removes the alignment boundaries of rKA, remains controversial due to concerns regarding reproducibility, implant safety, and long-term survivorship, particularly in knees with extreme preoperative alignment. Long-term outcome data across different knee phenotypes are still limited. This study addresses these uncertainties by evaluating mid- to long-term implant survivorship and patient-reported outcomes of urKA-TKA performed without alignment restrictions using a manual technique in a consecutive patient cohort.

The primary aim of this study is to present the mid- to long-term survival and functional outcomes of an non-designer series of urKA-TKA. The secondary aim is to assess the impact of urKA on implant survival and functional outcomes in different knee phenotypes. The tertiary aim is to assess if these outcomes and implant survival are different when urKA is employed outside the recommended boundaries for restricted KA.

## Methods

We prospectively reviewed a single-surgeon series of consecutive urKA-TKAs performed between March 2014 and October 2019 using the GMK Sphere knee system (Medacta International, Switzerland). All procedures were performed using a calipered measured resection technique without routine primary patellar resurfacing.

A total of 208 patients (229 knees) undergoing primary urKA-TKA were included and evaluated clinically and radiologically. There were no boundary restrictions regarding preoperative limb alignment or joint line obliquity. Patients with incompetent collateral ligaments and/or a history of ipsilateral osteotomy were excluded.

The surgical technique aimed to restore each patient’s pre-arthritic joint line and limb alignment, with no restrictions on the degree of preoperative varus, valgus, or flexion deformity. Ligament releases or lateral release were not performed, reflecting the underlying philosophy of KA. A standard medial parapatellar approach was used in all cases.

UrKA-TKA was performed manually using the calipered ‘measured resection’ technique,^13^ with excision of the posterior cruciate ligament and implantation of a cemented medial pivot prosthesis (GMK Sphere; Medacta International, Switzerland). All surgeries were performed by the same surgeon (DJ). The patella was not routinely resurfaced but was circumferentially denervated using electrocautery. One patient (one knee) underwent primary patellar resurfacing due to symptomatic patellofemoral osteoarthritis as the primary indication for TKA.

**Fig. 1:**
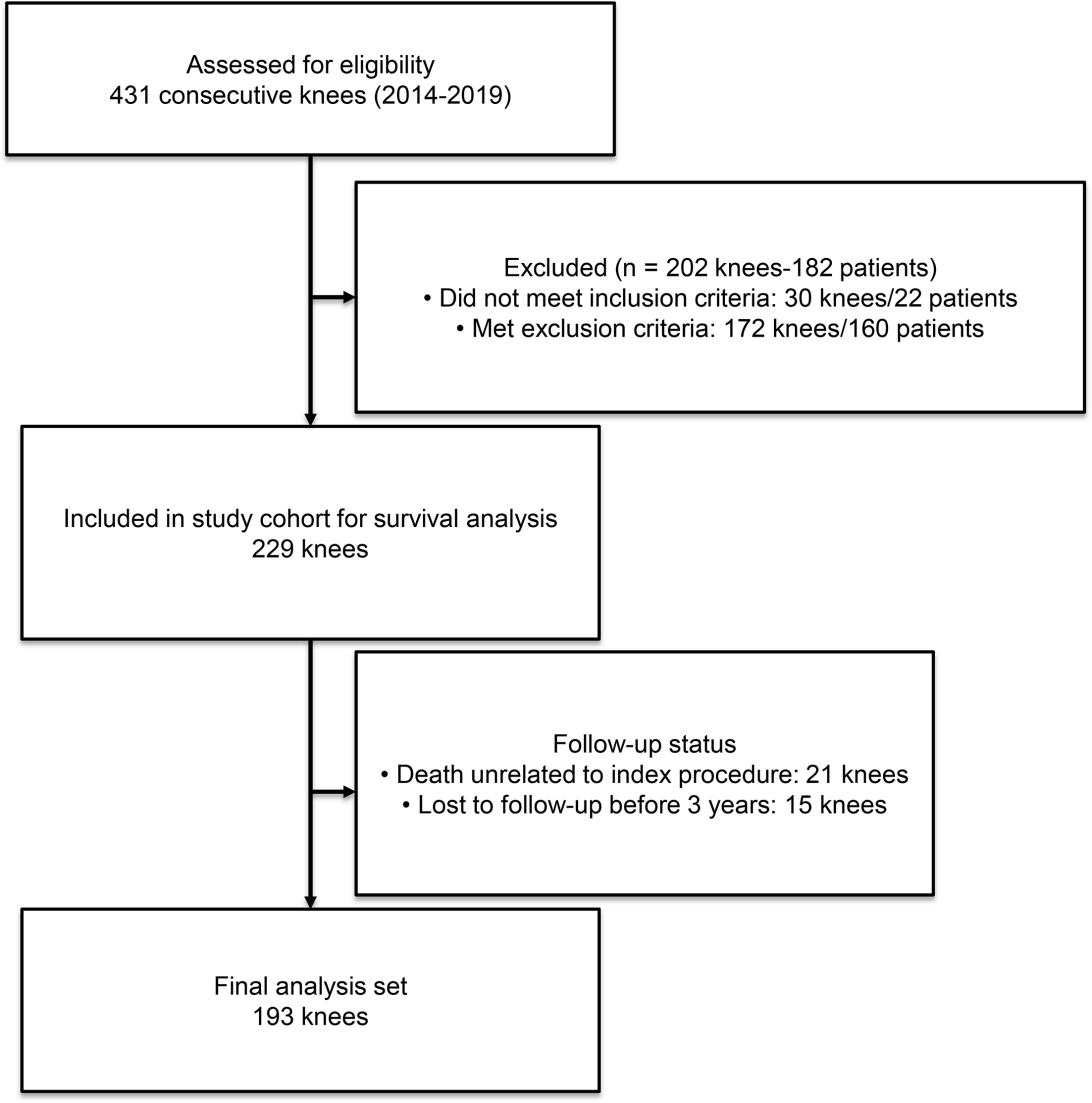
Study flowchart showing the number of knees included in the final analysis.

Patient demographic data including age, BMI, and sex were collected. During the study period, 19 patients (21 knees, 9.1%) died from causes unrelated to the index procedure, and 15 patients (15 knees, 6.6%) were lost to follow-up before 36 months. The mean follow-up was 87 months (36 to 136), with 193 knees followed for at least three years. A detailed flow diagram is reported in Figure 1.

### Patient characteristics

In total, 229 primary urKA TKAs were carried out in the study period: the mean age was 69.4 years (SD 9.5) and the mean was BMI was 31.5 kg/m² (SD 5.7).

Follow-up included face-to-face assessments with collection of clinical and radiological outcomes, including PROMs and standardized weightbearing long-leg radiographs.^14^ In addition, an attempt was made to contact all patients within the last six months to establish their current clinical status and collect PROMs when possible. For deceased patients, the general practitioner was contacted to determine whether any reoperation of the index knee had occurred prior to death; however, the absence of a centralized database limited the possibility of independent verification. For patients not traceable at final follow-up, the last date of physical or virtual contact and clinical status were recorded.

PROMs included the Forgotten Joint Score (FJS; 0 worst, 100 best),^15^ Knee Injury and Osteoarthritis Outcome Score (KOOS; 0 worst, 100 best),^16^ and the Numerical Rating Scale for pain (NRS; 0 no pain, 10 maximum pain).^17^ A patient acceptable symptom state (PASS) threshold of 33.3 for the FJS after primary total knee arthroplasty has been reported, while a score ≥ 77.1 has been proposed to indicate a ‘forgotten joint’ state.^18^

**Fig. 2:**
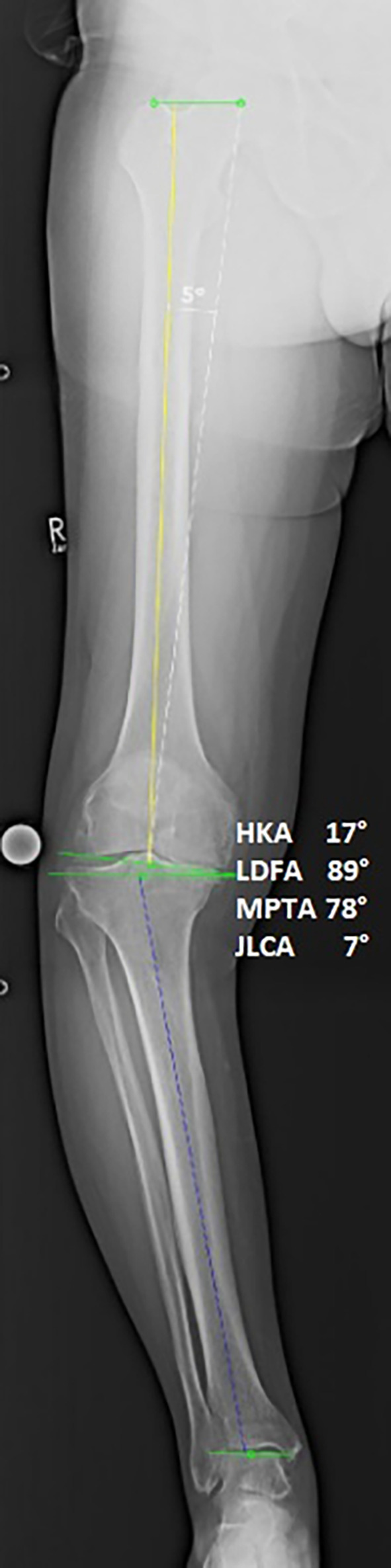
Preoperative full weightbearing long-standing anteroposterior radiograph of a varus knee with alignment references. HKA, hip-knee-ankle angle; JLCA, joint line convergence angle; LDFA, lateral distal femural angle; MPTA, medial proximal tibial angle.

**Fig. 3:**
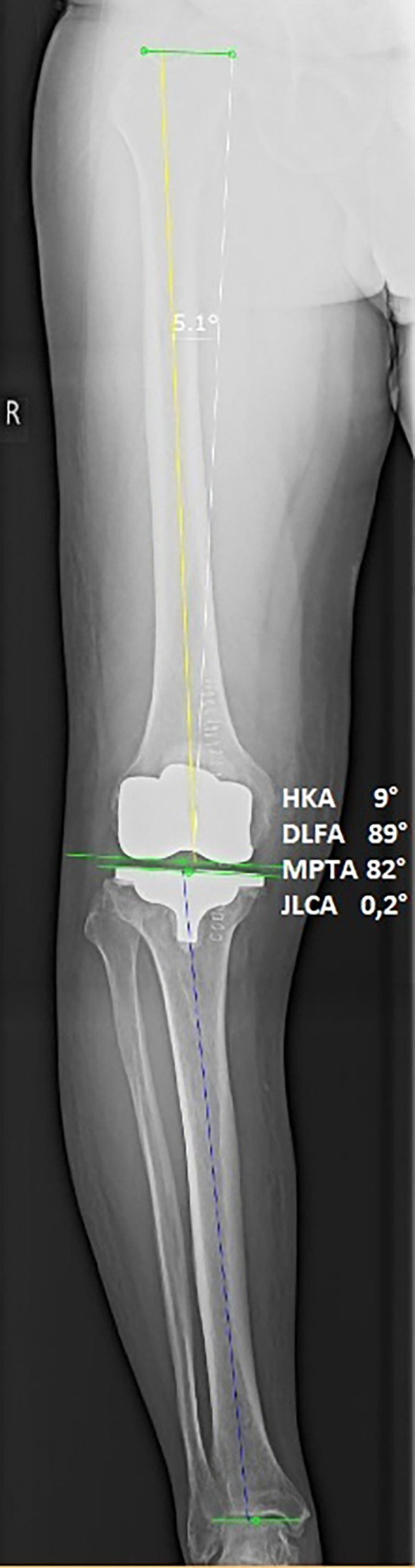
Postoperative full weightbearing long-standing anteroposterior radiograph of the knee in Figure 2 with alignment references. HKA, hip-knee-ankle angle; JLCA, joint line convergence angle; LDFA, lateral distal femural angle; MPTA, medial proximal tibial angle.

Preoperative and one-year postoperative weightbearing long-leg radiographs were used to assess lower limb coronal alignment. Alignment parameters were measured as angles relative to the vertical axis, with valgus angles added to and varus angles subtracted from 180°. Two blinded observers independently measured the hip–knee–ankle (HKA) angle, lateral distal femoral angle (LDFA), and medial proximal tibial angle (MPTA) (Figure 2 and Figure 3).

Arithmetic HKA and joint line obliquity were calculated to classify knees into nine phenotypes according to the Coronal Plane Alignment of the Knee (CPAK) classification.^19^ Lower limb alignment was categorized as neutral (0° ± 5°), varus (< −5°), or valgus (> + 5°) using the HKA angle, to identify patients who would have been considered unsuitable for unrestricted KA under a restricted KA philosophy. Severe deformities were defined as deviations greater than 10° from neutral alignment.

Implant survival was calculated using revision for any reason and revision for aseptic reasons as endpoints. Additional reoperations were also recorded. Revision was defined as the removal, exchange, or addition of any implant component.^20^

Sub-group analyses were performed to further explore the impact of preoperative alignment on clinical and radiological outcomes. First, patients were stratified according to the magnitude of preoperative coronal alignment into two groups: knees with neutral preoperative HKA within 5° compared with outliers. A second sub-group analysis compared outcomes among knees within and outside restricted boundaries and finally a third sub-group analysis included those with severe deformity in varus or valgus against those within 10° from HKA. A further analysis has been added to compare outcomes among knees within and outside the boundaries proposed by Bellemans et al^21^ (6° varus to 3 ° valgus for the HKA) to capture a wider range of constitutional phenotypes.

This study was designed as a prospective clinical audit conducted in accordance with institutional policies, approved by Institutional Review Board (P02.010.18) and regular updates provided as per board’s guidelines. All data were anonymized prior to analysis, and no additional diagnostic or therapeutic procedures outside standard clinical care were performed.

### Statistical analysis

Sample size considerations were based on implant survivorship as the primary outcome. Assuming an expected revision rate of ≤ 5% at mid-term follow-up, approximately ten revision events would be required to provide a reasonable descriptive estimate of implant survivorship. Under these assumptions, a sample size in the order of 200 knees would allow estimation of survivorship with an expected precision of approximately ± 3% around the survival estimate (95% CI). This sample size was considered adequate for descriptive survival analysis but not for powered comparisons between sub-groups, which were therefore regarded as exploratory.

Implant survivorship was assessed using the Kaplan-Meier analysis with 95% CIs with revisions and reoperations for any reason as endpoints. Variables were described with mean and SD or median and IQR, if not normally distributed. Paired and unpaired *t*-test and Mann-Whitney U-test were used as appropriate to compare continuous variables. Chi-squared test and Fisher’s exact test were used for categorical variables. A p-value < 0.05 was considered as statistically significant. Shapiro-Wilk tests were performed to test the datasets for distribution and appropriate statistical analyses performed. JASP (Jeoffrey’s Amazing Statistics programme, Netherlands) software was used for the statistical analysis.

## Results

### Survivorship outcomes

Revision (6 knees/229, 2.6%) and reoperation (2 knees/229, 0.9%) surgeries are reported in Table I.

**Table I.** Revision and reoperatorion surgeries rate.

Surgeries	N (%)
Secondary patellar resurfacing	4 (1.7)
Revision for PJI	1 (0.4)
Revision for periprosthetic fracture	1 (0.4)
Artrhroscopic debridement and MUA	1 (0.4)
Arthroscopic debridement for effusions	1 (0.4)
Total	8 (3.4)

MUA, manipulation under anaesthesia; PJI, periprosthetic joint infection.

**Fig. 4:**
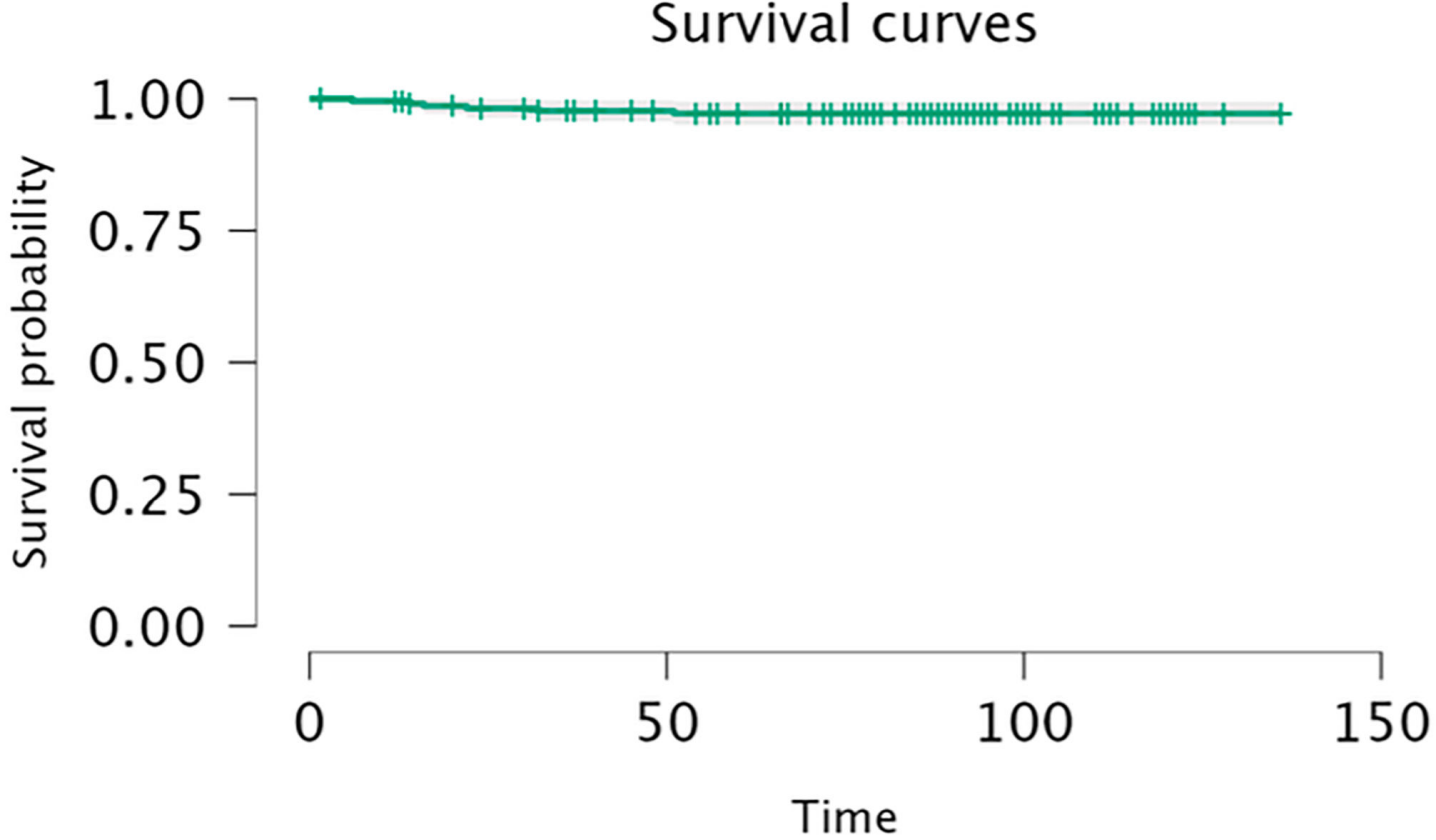
Kaplan-Meier survival curve with revision as endpoint (time expressed in months).

At the 8.8 years of follow-up (32 at risk), implant survival with revision (n = 6) and any reoperation (n = 8) as endpoints were 97.2% (95% CI 95.0 to 99.4; Figure 4 and Table II) and 96.3% (95% CI 93.9 to 98.9), respectively. For aseptic revisions, implant survival was 97.7% (95% CI 95.7 to 99.7). There were no revisions for aseptic loosening (implant survival 100%). No significant difference in terms of implant survival (five events, excluding septic reason) were found between valgus knees (CPAK III, VI and IX) compared with neutral and varus knees (p > 0.05) and between neutral (within ± 5° boundaries) and outliers (p > 0.05). No significant difference in terms of implant survival were found (p = 0.645) between knees inside and outside preoperative restricted alignment boundaries of ± 3° from neutral for HKA and of ± 5° from neutral for JLO (survival 96.4% (21 knees at risk, 95% CI 89.8 to 100.0) vs 97.9% (140 knees at risk, 95% CI 95.8 to 100.0)). Considering postoperative restricted alignment boundaries, no significant difference in terms of implant survival were found (p = 0.446) 98.7% (26 at risk, 95% CI 96.2 to 100.0) vs 97.1% (48 at risk, 95% CI 94.3 to 99.9)). No significant difference in terms of implant survival (p = 0.732) were found in patients with severe deformities (> 10°) with a 97.7% (34 at risk, 95% CI 93.3 to 100) for severe varus and 100% (11 at risk, 95% CI 100 to 100) for severe valgus against 96.8% (116 at risk, 95% CI 94.0 to 99.6) for non-severe deformities (-10° to 10°).

**Table II.** Life table with revision as endpoint and patient-at-risk at each follow-up time.

Time, mtns	At risk	Events	Survival	Standard error	95% CI
2.000	227	0	1.000	0.000	1.000 to 1.000
16.000	215	3	0.986	0.008	0.971 to 1.000
31.000	209	1	0.982	0.009	0.964 to 1.000
46.000	199	1	0.977	0.010	0.957 to 0.997
61.000	191	1	0.972	0.011	0.950 to 0.994
76.000	161	0	0.972	0.011	0.950 to 0.994
91.000	74	0	0.972	0.011	0.950 to 0.994
106.000	32	0	0.972	0.011	0.950 to 0.994
121.000	11	0	0.972	0.011	0.950 to 0.994
136.000	1	0	0.972	0.011	0.950 to 0.994

At the 8.8 years’ follow-up (21 at risk), implant survival for knees with preop. HKA outside 6° varus and 3° valgus with revision (n = 5) and any reoperation (n = 5) as endpoints were both 96.4% (95% CI 93.3 to 99.5). No significant difference in terms of implant survival were found for revisions (p = 0.306) and reoperations (0.974) between knees inside and outside these boundaries (6° varus to 3° valgus).

### Patient-reported outcomes

All functional outcomes showed substantial improvement and are reported in Table III. Pain scale scores at the last follow-up were a mean of 1.6 (SD 2.4) on the NRS, with a median of 0 with most patients (105/175, 60.0%) reporting no pain (Table III). No significant differences in terms of postoperative KOOS (p = 0.497), three years FU FJS (p = 0.495), latest FU FJS (p = 0.448), and latest follow-up NRS (p = 0.505) were found among knees respecting both preoperative restricted boundaries of ± 3° from neutral for HKA and of ± 5° from neutral for JLO. No significant differences were found for those respecting the boundaries in the postoperative alignment as well: postoperative KOOS (p = 0.589), three years follow-up FJS (p = 0.474), latest follow-up FJS (p = 0.297), and latest follow-up NRS (p = 0.539).

**Table III.** Clinical and radiological outcomes of overall assessment.

Variable	Mean preoperative (SD), (number of knees)	Mean postoperative (SD), (number of knees)	p-value
KOOS	28.8 (10.3) (229)	75.8 (16.6) (229)	p < 0.001
FJS (3 year FU)		67.5 (25.8) (229)	
FJS (last FU)		87.6 (17.7) (175)	p < 0.001
Pain (last FU)		1.6 (2.4) (175)	
HKA angle	−3.0° (7.9°) (229); (range −20.4° to 25.2°)	0.2° (4.0°) (229); (range −9.4° to 10.6°)	p < 0.001

FJS, Forgotten Joint Score; FU, follow-up; HKA, hip-knee-ankle; KOOS, Knee Injury and Osteoarthritis Outcome Score.

For preoperative HKA outliers, significant differences were found in postoperative KOOS with an improvement from a median of 27.9 and 29.3 (p = 0.220) to 72.6 and 77.6 (p = 0.007) for patients within and outside ± 5° range, respectively. Similar results were noted for these outliers with significantly better FJS with 62.5 and 82.2 points respectively at three years and latest follow-up for patients within ± 5° boundaries and 70.4 and 91.0 for those beyond (p = 0.013 at three years follow-up and p = 0.008 at the latest follow-up). No significant differences were found for KOOS, ΔKOOS, or FJS scores (p > 0.05) between CPAK categories.

A PASS threshold of 33.3 for the FJS at three years follow-up was reached in 89.6 knees (173/193), while a ‘forgotten joint’ state (with a score ≥ 77) was reported in 47.7% knees (92/193).

### Radiological outcomes

**Fig. 5:**
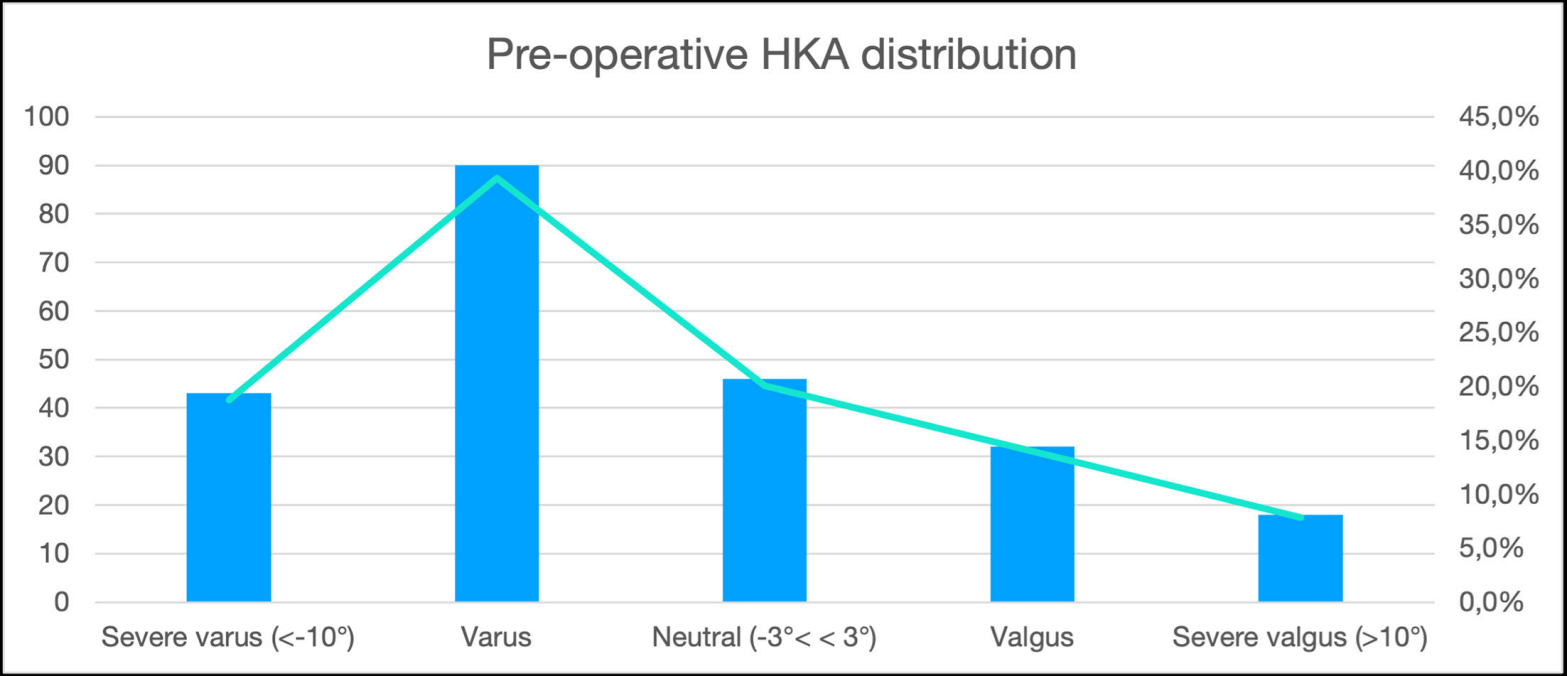
Preoperative hip-knee-ankle (HKA) alignment distribution across varus, neutral, and valgus categories.

**Fig. 6:**
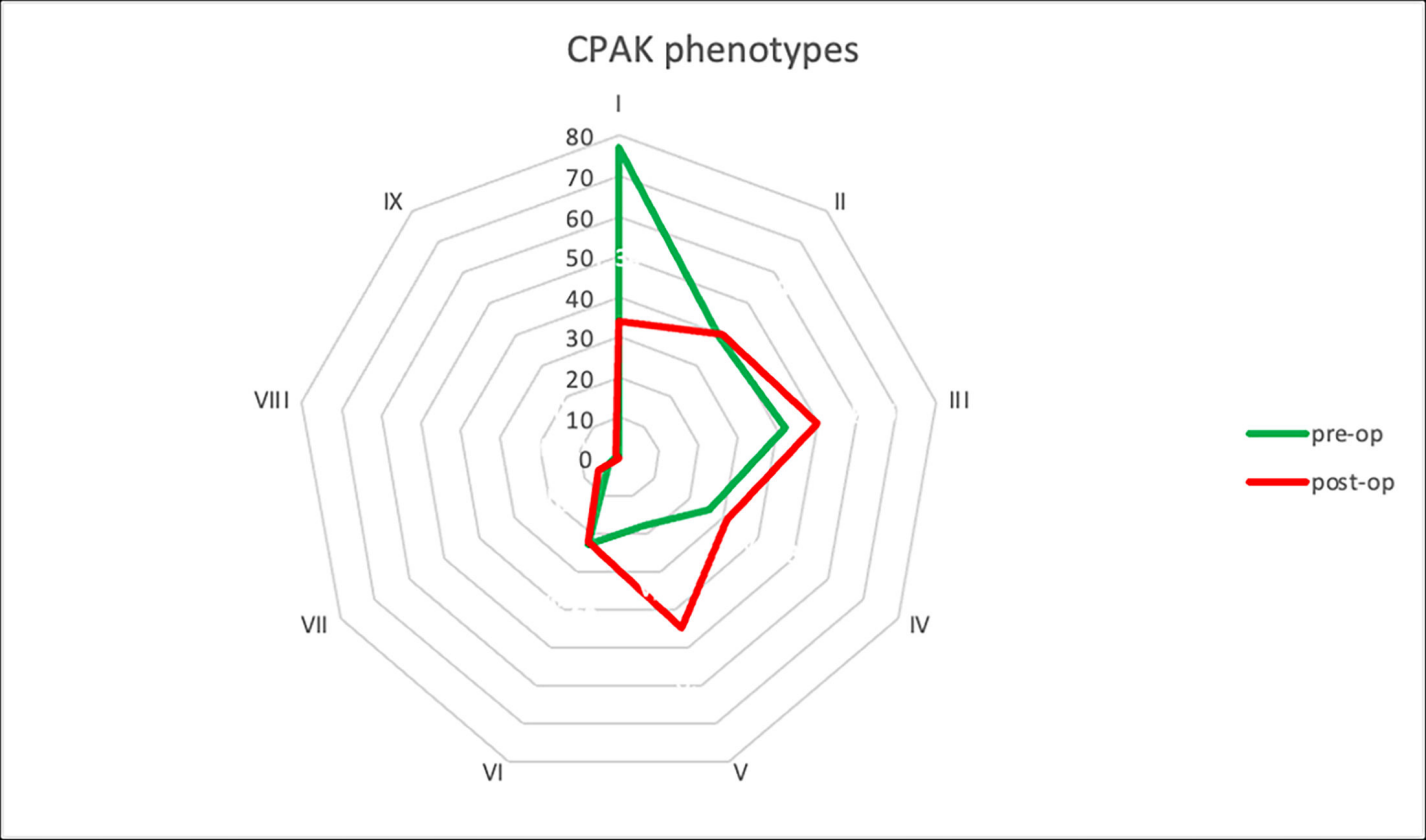
Spider diagram showing Coronal Plane Alignment of the Knee (CPAK) phenotypes change from preoperative to postoperative.

**Fig. 7:**
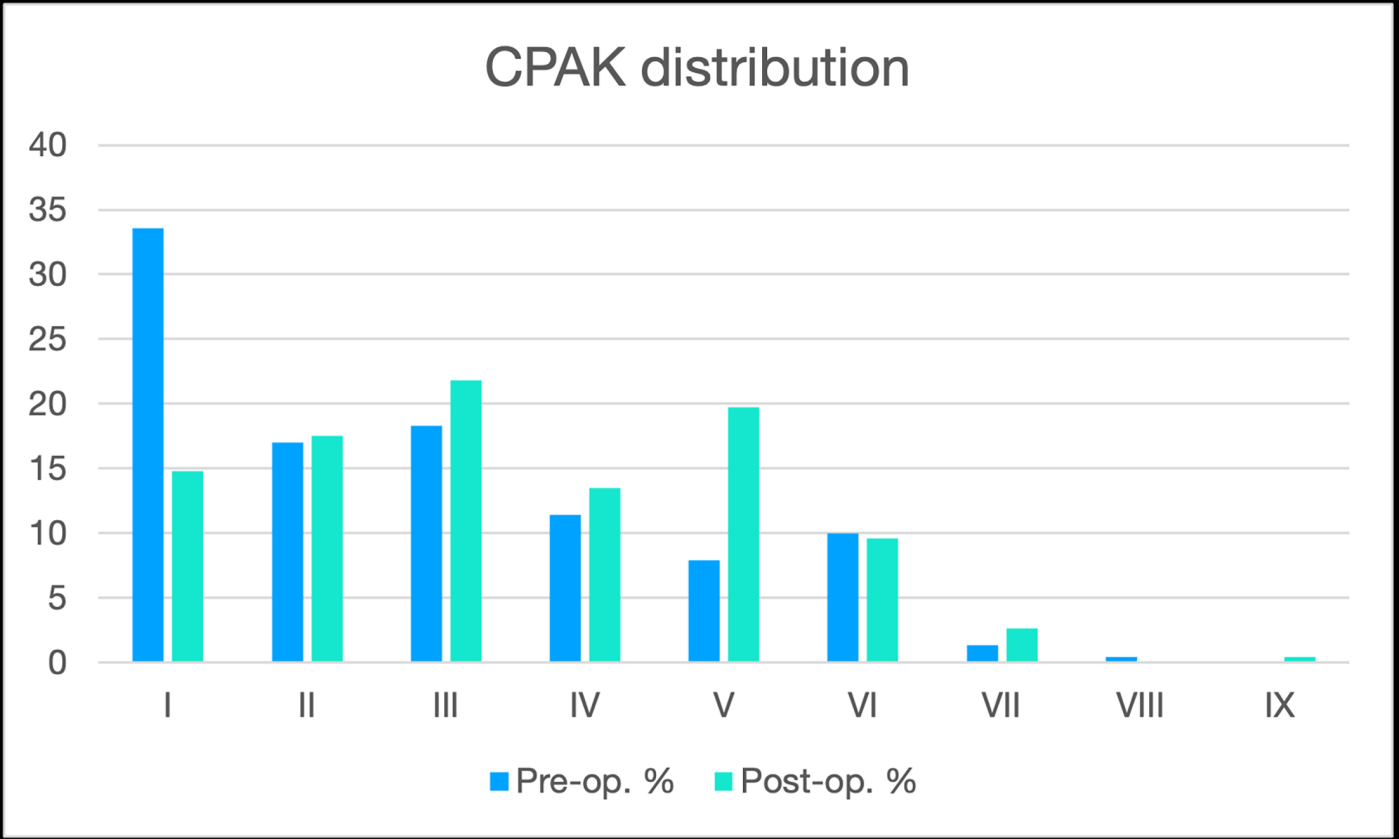
Preoperative and postoperative Coronal Plane Alignment of the Knee (CPAK) distribution.

The mean preoperative and postoperative HKA angles are reported in Table III. Preoperatively only 30 knees (13.1%) respected the restricted alignment boundaries (± 3° from neutral HKA and of ± 5° from neutral JLO). Knees with severe varus (range 10.0° to 20.4°) and severe valgus (range 10.3° to 25.2°) were 43 (18.8%) and 18 (7.8%), respectively (Figure 5). CPAK did not change in 75 knees (32.8%) with the phenotype modification mainly due to an increase in MPTA (p < 0.001) with a stable LDFA (p = 0.912). The most frequent changes in CPAK were one step to the right (43/229, 18.8%) and one step down (37/229, 16.2%). A change towards apex proximal groups (CPAK VII, VIII, IX) was rare (6/229, 2.6%) (Figure 6 and Figure 7).

### Secondary patella resurfacing

No significant associations were found between patellar reoperation rates and preoperative HKA ± 5° outlier status (p = 0.613), sex (p = 0.448), or postoperative CPAK classification (p = 0.062).

Four patients (4/228, 1.8% of the cohort, primary patellar resurfacing excluded) underwent secondary patella resurfacing with two out of four being in the CPAK III, VI or IX groups (2/64, 3.1% vs 2/164, 1.2%). No statistically significant difference was observed between groups (odds ratio 2.61, 95% CI 0.36 to 18.96; p = 0.310).

## Discussion

This study demonstrates that TKA performed using unrestricted kinematic alignment provides excellent mid- to long-term implant survivorship and significant improvements in PROMs. It includes the learning curve of a non-designer surgeon with a single implant and manual instruments without the use of any assistive technology. No patients were excluded due to the severity of preop deformity provided the collateral ligaments were intact.

Over a nine-year follow-up, survivorship was > 97% for revision and > 96% for reoperation, with no aseptic loosening recorded. Functional improvements in the KOOS and FJS were both statistically and clinically significant, largely exceeding minimal important change differences and showing progressive improvement over time.^22^ The FJS is particularly relevant in this context and KA-TKA seems to succeed in its aim of restoring a more natural knee, thus ‘forgetting’ the artificial joint during daily activities. Recently, statistical, but not clinical, significant improvement in KSS Pain score, Western Ontario and McMaster Universities Osteoarthritis Index (WOMAC),^23^ and FJS have been found at two years for medial-pivot urKA-TKA compared with MA-TKA, especially in patients with varus knee.^23^ This clinical irrelevance has been confirmed in a recent meta-analysis by Migliorini et al^7^ comparing kinematic and mechanical alignment.

Furthermore, outcomes were not compromised when urKA was performed in patients with knees outside the traditionally recommended rKA boundaries,^4^ suggesting that restoring each patient’s native joint line and limb alignment is both successful and beneficial across a wide spectrum of phenotypes. Interestingly, patients with a far non-neutral preoperative alignment (>± 5° from neutral) demonstrated greater improvements in KOOS and FJS than those within the neutral boundaries. This finding suggests that patients with atypical anatomies benefit more from a personalized technique which aims to restore their native alignment, rather than enforcing mechanical neutrality. This is not surprising as recently comparable clinical outcome enhancements were described also for severe varus knee (> 10°) compared with patients with mild varus.^24^ Same satisfying clinical results without increasead complications at a minimum two years follow-up have been reported by Bar Ziv et al^10^ for valgus knees.

**Fig. 8:**
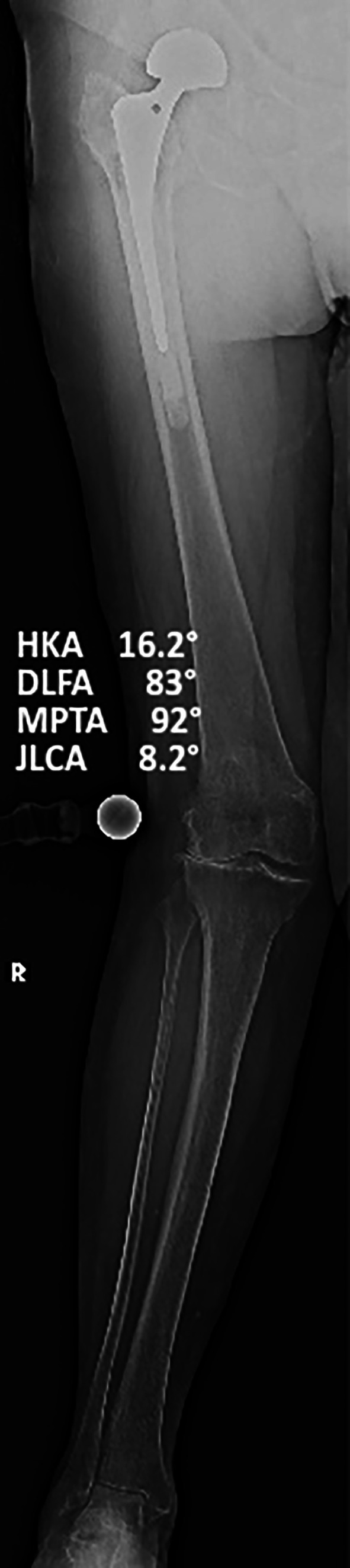
Preoperative full weightbearing long-standing anteroposterior radiograph of a severe valgus knee with HKA of 16.2°. HKA, hip-knee-ankle angle; JLCA, joint line convergence angle; LDFA, lateral distal femural angle; MPTA, medial proximal tibial angle.

Severity of deformity, both in valgus (18 knees, max valgus 25.2°, Figure 8) and in varus (43 knees, max varus 20.4°), does not compromise survival, supporting the fact that restricted boundaries could be too prudent.

Howell et al^25,26^ showed excellent survival results of 97.5% for revision for any reason and 98.4% for aseptic failure at ten years^25^ and 93.0% at 16 years.^26^ In an observational study from the Australian and New Zealand Joint arthroplasty Registries, good-to-excellent results with a cumulative revision rate at seven years of 3.1%, similar to those of computer-assisted surgery and conventionally instrumented TKA, have been also reported for urKA-TKA performed using patient-specific instrumentation.^27^ Our results confirm and support these original series with excellent survivorship of 97% free from revision for any reason at nine years, and 100% survivorship for aseptic loosening, using the calipered measured resection technique. This is the first non-designer study reporting on mid- to long-term survival and clinical outcomes. The implant survivorship reported in our series is similar to that reported by Morcos et al (99% at 11.3 years, n = 104, navigated rKA-TKAs)^28^ with one revision for instability. In our cohort, if Vendittoli boundaries were applied, only 30 patients (13.1%) would be eligible for KA.

In all personalized alignment approaches (except urKA), surgeons advocate the use of assistive technology with aim of ensuring the implant and leg alignment are within a prespecified boundary. These change from one philosophy to another and necessitate the use of robotic assistance. UrKA can be consistently performed using manual instruments and one can obtain excellent survivorship without adoption of any complex algorithms. The absence of aseptic loosening in our series is particularly noteworthy also because no boundaries were considered in those urKA-TKA. These results provide reassuring evidence that restoring native alignment does not increase mechanical failure risk, at least within the follow-up duration studied.

CPAK phenotypes change in about 67% of patients mainly due to an increased MPTA, probably to cope with bone defects on medial tibial plateau. Similar observations were made by Morcos et al^28^ when they reported on their series of rKA.^28^ This phenotype change could also arise from a measurement bias as the 2D coronal analysis cannot take into consideration rotational and flexion deformity.^29^ Furthermore, in severe arthritis, the reference points’ identification to draw the alignment could be challenging.

The patello-femoral joint (PFJ) needs a special mention. Although KA aims to restore the native Q-angle and better reproduce the native trochlear anatomy, patellar complications may represent an issue with urKA.^30^

In our series, valgus phenotypes (CPAK III, VI, and IX) showed higher odds of patellar complications (OR 2.61) as previously stated by Howell et al^31^ and Lustig et al;^32^ however, this finding did not reach statistical significance, likely due to the limited number of events.

While the overall risk remains low, careful PFJ assessment is fundamental for a well-working pain-free TKA. Routinely available implants (originally developed with MA philosophy in mind) have a 6^°^ valgus prosthetic trochlear angle (PTA) relative to the mechanical axis aimed at optimizing patellar tracking. The risk with KA is a medial deviation of the groove, especially in valgus knees relative to the femoral mechanical axis. Providing implants specific for KA with a higher PTA could help reduce the risk of PFJ issues with the valgus phenotypes.

The key strengths of this study are its large sample size, consecutive patients, consistent surgical technique, and use of a single implant type, which reduces variability. The mid- to long-term follow-up provides robust evidence of implant durability and functional sustainability. It also includes the learning curve and does not have any exclusion criteria (based on preop deformity). The single surgeon series is a strength of the study but on the other hand may limit external validity. We also acknowledge potential variation in the learning curve as well as extent of coronal plane deformities based upon where a surgeon practices. The lack of a control group makes it impossible to draw definitive conclusions about the best alignment to aim to. Lack of long-term radiological data could underestimate the loosening rate as one can’t account for asymptomatic loosening.

A limitation of this study is that information on surgical procedures prior to death was obtained through contact with the patients’ general practitioners, which may not have captured all interventions and therefore may be subject to incomplete reporting.

Furthermore, no correction for multiple testing was applied; therefore, the reported p-values may be affected by the risk of multiplicity, increasing the likelihood of false-positive findings.

This study has confirmed that manual urKA-TKA is a clinically successful and effective technique with excellent mid- to long-term outcomes. Importantly, outcomes were not compromised in presence of a severe deformity, provided collateral ligaments are competent. On the contrary, patients with more pronounced deformities demonstrated greater functional improvements than those with near-neutral alignment, supporting the principle of restoring native anatomy rather than enforcing mechanical neutrality. Patellofemoral complications represent an issue particularly in valgus phenotypes. Although rare, these need a careful assessment and possibly adoption new implant designs.

**Take home message**

- Unrestricted kinematic alignment-total knee arthroplasty is a clinically successful and effective technique, which enhances the potential for excellent mid- to long-term outcomes.

- Clinical and survival results are not influenced by the extent of preoperative alignment, provided the collateral ligaments are intact.

## Data Availability

The datasets generated during and/or analyzed during the current study are available from the corresponding author on reasonable request.
